# Antimicrobial activity of ZnO-Ag-MWCNTs nanocomposites prepared by a simple impregnation–calcination method

**DOI:** 10.1038/s41598-023-48831-w

**Published:** 2023-12-05

**Authors:** Rashad Al-Gaashani, Mujaheed Pasha, Khadeeja Abdul Jabbar, Akshath R. Shetty, Hussein Baqiah, Said Mansour, Viktor Kochkodan, Jenny Lawler

**Affiliations:** 1 Qatar Environment and Energy Research Institute (QEERI), Hamad Bin Khalifa University (HBKU), Qatar Foundation, 34110 Doha, Qatar; 2 HBKU Core Labs, Hamad Bin Khalifa University, Qatar Foundation, Doha, Qatar; 3 Shandong Key Laboratory of Biophysics, Institute of Biophysics, Dezhou University, No.566 University Rd. West, Dezhou, Shandong China

**Keywords:** Biochemistry, Biological techniques, Materials science, Nanoscience and technology

## Abstract

Zinc oxide (ZnO) nanorods and ZnO nanostructures composited with silver (Ag) and multi-walled carbon nanotubes (MWCNTs) have been synthesized by a simple impregnation–calcination method and have been shown to be suitable for use as antimicrobial agents. The preparation method used for composite materials is very simple, time-effective, and can be used for large-scale production. Several analytical techniques, including X-ray diffraction (XRD), scanning electron spectroscopy (SEM), energy dispersive X-ray spectroscopy (EDS), transmission electron microscopy (TEM) and Fourier transmission infrared spectroscopy (FTIR), have been used to characterize the prepared ZnO-Ag-MWCNT composite materials. The effects on structural, morphological, and antimicrobial properties of (ZnO)_100-x_ (Ag)_x_ nanocomposites at various weight ratios (x = 0, 5, 10, 30, and 50 wt%) have been investigated. The antimicrobial properties of ZnO composited with Ag nanoparticles and MWCNTs towards both gram-positive and gram-negative bacteria species were studied. The effect of raw MWCNTs and MWCNTs composited with ZnO and Ag on the cell morphology and chemical composition of Staphylococcus aureus (*S. aureus*) and Escherichia coli (*E. coli*) was studied by SEM and EDS, respectively. It was found that composite materials made of ZnO-Ag-MWCNTs exhibit greater antibacterial activities toward the microorganisms *E. coli* and *S. aureus* than ZnO-Ag, which could be beneficial for efficient antimicrobial agents in water and air treatment applications.

## Introduction

Research on nanomaterials as antimicrobial agents has become an interdisciplinary link between physicists, chemists, biologists, and medicine. Metal nanoparticles, metal oxide nanostructures, and carbon nanotubes (CNTs) are mainly used for improving the antimicrobial properties of polymer materials for food packaging^1,2^. Adding nanomaterials to food surfaces to stop bacterial growth and employing nanomaterials in intelligent packaging materials are the main benefits of using nanomaterials in food nanotechnology^3^. Silver (Ag) and zinc oxide (ZnO) nanostructures are commonly used for antimicrobial applications^4–7^. ZnO is utilized as an antibacterial agent in food packaging and against foodborne pathogens in the food industry. Additionally, ZnO nanostructures may largely stop the growth of fungi, gram-positive bacteria, and gram-negative bacteria, which reduces the danger of cross-contamination and increases product shelf life. The primary antimicrobial mechanism is usually based on the generation of reactive oxygen species (ROS), which can vary depending on the size and morphology of ZnO nanostructures^8^.

The Ag-ZnO nanocomposites have attracted much interest from researchers because it has biological and photocatalytic activity, low toxicity, and a synergistic antibacterial effect. Ag-ZnO nanocomposite materials have been suggested to be used in various applications, such as antimicrobial agents^4–7^, sensors^9–13^, solar cells^14,15^, and photo-catalysts^16–21^. Ag nanoparticles exhibit better antibacterial activity than ZnO nanostructures. However, ZnO-Ag nanocomposites can effectively inhibit the bacteria because of the interaction between Ag and ZnO. On the other hand, the cytotoxicity and genotoxicity of Ag nanoparticles on human cells have been reported^22–24^. Doping Ag with ZnO makes Ag nanoparticles safe to use since the amount of Ag can be successfully reduced after doping^25^. One of the key advantages of using ZnO-Ag nanocomposites for water treatment is their ability to effectively remove bacteria and other microorganisms from water. Bacterial contamination can cause serious health problems, and the presence of bacteria in drinking water is a major concern in many communities. The unique properties of ZnO-Ag nanocomposites make them highly effective at killing bacteria and other microorganisms, reducing the risk of exposure to these harmful contaminants. Single-walled carbon nanotubes (SWCNTs) and multi-walled carbon nanotubes (MWCNTs) were tested for their antibacterial efficacy against *E. coli*, showing that size is an important factor and SWCNTs are much more toxic to bacteria than MWCNTs^26^. Kang et al*.*^27^ reported that functionalized MWCNTs that are unbundled, uncapped, short, and disseminated in solution exhibit greater bacterial cytotoxicity than SWCNTs. The most common gram-positive and gram-negative bacterial species that cause many human diseases are *Escherichia coli* (*E. coli*) and *Staphylococcus aureus* (*S. aureus*), respectively.

In this study, our main objective is to examine the effect of ZnO nanostructures composited with silver and carbon nanotubes in different ratios on bacterial activity, namely *E. coli* and *S. aureus* bacteria. The morphology, structure, and growth mechanism of prepared materials were also studied in detail. The prepared nanocomposite materials could be used as promising materials with antibacterial properties in water treatment applications.

## Experiments

### Materials

Zinc acetate dihydrate (Zn(CH_3_COO)_2_ × 2H_2_O, 99.999 wt%), silver nitrate (AgNO_3_, 99 wt%), multiwall carbon nanotubes (MWCNTs, > 98 wt%), and nitric acid (HNO_3_) (67–69%, RomilSpA limited™, UK) were all purchased from Sigma Aldrich. Aqueous solutions were prepared using deionized water (DIW) with a conductivity of 18.2 MΩ/cm and nitric acid. Mueller–Hinton agar (Sigma) and petri dishes (Fisherbrand) were used for antibacterial testing. The agar plates were washed using DIW and autoclaved for 15 min at 121 °C.

### Preparation of ZnO and ZnO-Ag nanocomposites

ZnO and ZnO **c**omposited with Ag samples were prepared by a simple heating method. ZnO and ZnO-Ag composite samples were prepared by rapid heating of zinc acetate dehydrate and silver nitrate in a muffle furnace (Thermo Scientific Thermolyne 5.8L A1 Benchtop Muffle Furnace, 240V) under air at 400 °C for 60 min. In a typical experiment, 3 g of (zinc acetate dehydrate)_100-X_ and (silver nitrate)_x_ at various weight ratios (x = 0, 5, 10, 30, and 50 wt%) were mixed and dissolved in 10 ml of deionized water (5 min of stirring and 5 min of sonication in an ultrasonic bath). The mixed solutions were placed in alumina crucibles and heated for 60 min at 400 °C. When the furnace was shut off, the ZnO and ZnO-Ag composite samples were taken out and allowed to cool to ambient temperature.

### Preparation of ZnO-Ag-MWCNTs composites

MWCNTs composited with 40 wt% ZnO and 10 wt% Ag sample was also synthesized by a simple impregnation method. In a typical experiment, 1.2 g of zinc acetate dihydrate and 0.3 g of silver nitrate were mixed and dissolved in a mixture of solvents (10 ml deionized water and 5 ml nitric acid). The mixture was impregnated onto 1.5 g of MWCNTs, stirred for 15 min and sonicated in an ultrasonic bath for 15 min. The impregnated materials were then placed inside an alumina crucible and calcined for 60 min at 400 °C. The sample was then taken out after the furnace was shut off and allowed to cool to room temperature. The produced samples were washed with deionized water many times (pH about 7) and dried at 80 °C for 24 h. By weighing the ratio of zinc acetate dihydrate to silver nitrate to MWCNTs, the weight percentage of various components in the final composite was determined.

### Antibacterial activity of prepared materials

*Escherichia coli* cultures were grown in Lysogeny broth (LB) overnight and re-cultured the next morning for four hours to reach the exponential phase. The suspension was centrifuged to get the pellet and re-suspended in phosphate buffered saline (PBS) solution. The *E. coli* culture was spread on the agar plate, and each material (ZnO + 5% Ag, ZnO + 10% Ag, ZnO + 30% Ag, ZnO + 50% Ag, MWCNTs + 40% ZnO + 10% Ag, and MWCNTs) was placed in a circle shape (10 mg each) on the bacterial culture plate. The plate was incubated at 37 °C for overnight to check the antibacterial activity against each material. The microorganism strain used in the antibacterial activity of *E. coli* gram-negative bacteria was NCTC 9001.

Staphylococcus aureus cultures were also grown in Lysogeny broth (LB) overnight and re-cultured the next morning for four hours to reach the exponential phase. The microorganism strain used in the antibacterial activity of *S. aureus* gram-positive bacteria was ATCC 23235. The suspension was centrifuged to get the pellet and re-suspended in phosphate buffered saline (PBS) solution. The *S. aureus* culture was spread on the MH (Meuller Hinton) agar plate, and each material (ZnO + 5% Ag, ZnO + 10% Ag, ZnO + 30% Ag, ZnO + 50% Ag, MWCNTs + 40% ZnO + 10% Ag, and MWCNTs) was placed in a circle shape on the bacterial culture plate. The plates were then incubated at 37 °C for overnight to check the antibacterial activity against each material. The plates were observed under a magnificent lens to see the zones of inhibition. The diameter of the zone of inhibition was determined around the material and recorded in mm for *E. coli* and *S. aureus* (Table 2).

### Samples characterization

The prepared materials were characterized by several advanced techniques. X-ray diffraction (XRD) (Bruker D8 Advance X-Ray Diffractometer with Cu-Kα radiation source) was used to study the structure of the synthesized materials. SEM/EDS characterization was performed using the JEOL JSM 7800F FE-SEM and the Oxford Instruments EDS detector: X-Max 80 mm^2^ as follows: Powder samples are sprinkled onto a double-sided adhesive carbon tape, and excess powder is blown off. For SEM imaging, a 5 kV acceleration voltage is used. An electron (BSE) imaging detector is used to show the atomic number contrast difference between Ag and ZnO. For EDS, a 10 kV acceleration voltage is used; this enabled us to limit the excitation volume to the powder sample and avoid signals from the carbon tape and aluminum stub. Using 10 kV excitation voltage-enabled signals from AgLα, ZnLα and OKα. The ZnKα line is not used as ZnLα gives a much better peak-to-background ratio at 10 kV excitation voltage. The Quanta650FEG SEM is also used for imaging and the Bruker Quantx400 EDS is used for microanalysis. The morphology of all samples was studied by FE-SEM and high-resolution transmission electron microscopy (HRTEM) using Thermo TalosX. Thermo Scientific Nicolet iS50 FT-IR spectrometer was used to analyze the samples' FTIR spectra in the transmittance mode, and the sample discs were made of KBr pellets. In the spectral range of 4000–400 cm^−1^ at a spectral resolution of 4 cm^−1^, the spectra were obtained with 32 scans per sample and background.

## Results and discussion

### Structure and morphology studies of the synthesized samples

**Figure 1:**
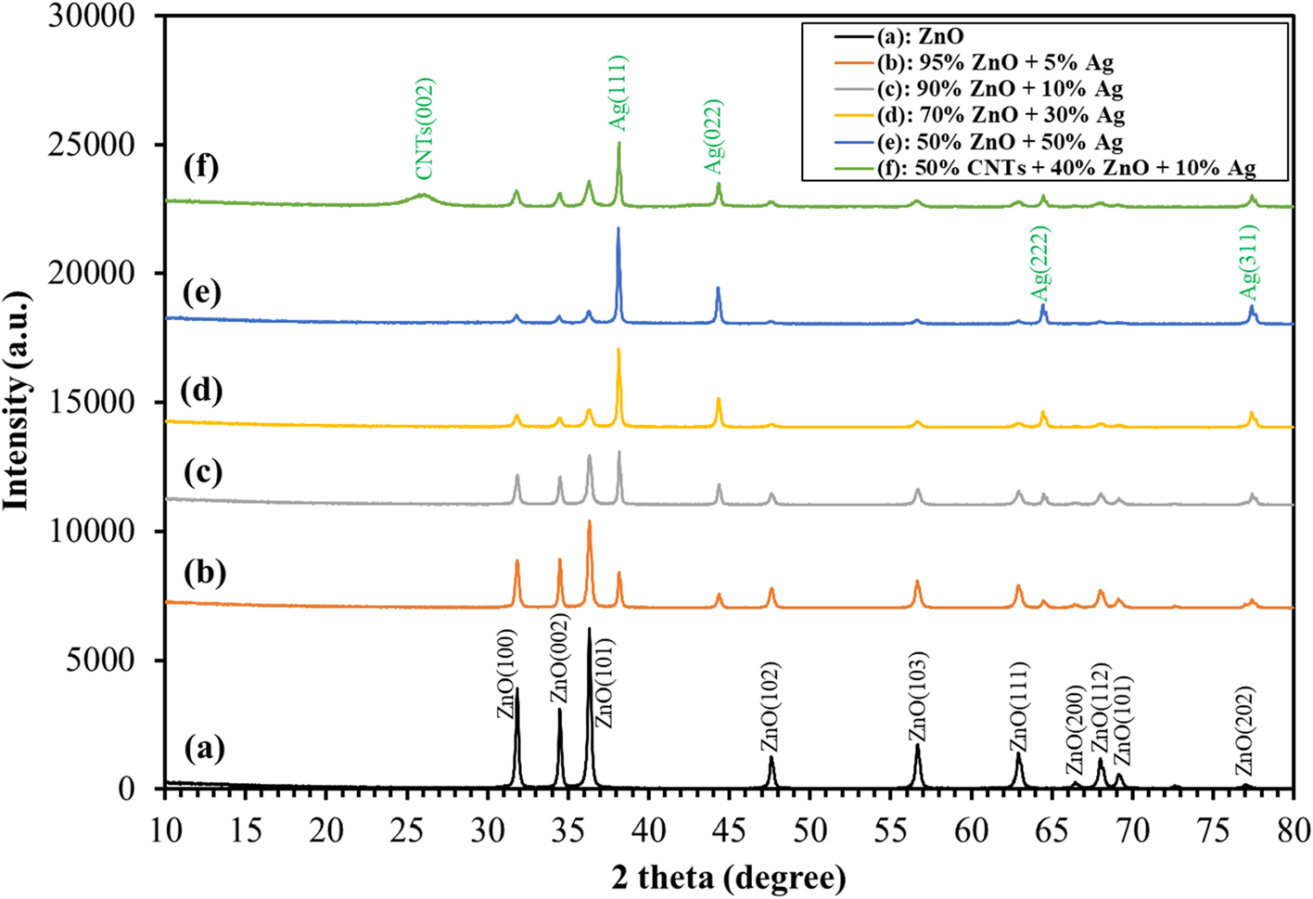
XRD patterns of ZnO nanorods (a); (b) 95 wt% ZnO + 5 wt% Ag; 90 wt% ZnO + 10 wt% Ag (c); 70 wt% ZnO + 30 wt% Ag (d); 50 wt% ZnO + 50 wt% Ag (e); and 50 wt% MWCNTs + 40 wt% ZnO + 10 wt% Ag (f).

The XRD patterns of ZnO and ZnO-composited Ag and MWCNTs with different ratios are displayed in Fig. 1. The peaks reveal that the material is crystalline in nature with no impurities. The ZnO peaks can be successfully indexed to the hexagonal phase with the wurtzite structure corresponding to JCPDS No. 01-082-9744. The peaks of the Ag matched up to the typical powder diffraction card of JCPDS No. 01-087-0717. From Fig. 1, the incorporation of Ag reduces the peak intensities of intense planes corresponding to ZnO (100), (002), and (101), associated with an increase in peak intensities of silver (111). The crystallite size of ZnO and Ag can be determined through the full width at half maximum (FWHM) of the XRD peak by using the Scherer equation. Table 1 displays the *d*-spacing as well as the crystallite size obtained from ZnO and Ag peaks. Table 1 clearly shows that the addition of Ag dopant influences a significant reduction in the crystallite size corresponding to ZnO and an increase in grain size for silver. Table 1 clearly shows that the presence of Ag dopant results in a large drop in the ZnO crystallite size and an increase in the silver grain size. Interestingly, we do not notice any change in the d-spacing for ZnO and Ag, despite Ag-doping. In Fig. 1f, the peaks corresponding to CNT (002) and Ag, in addition to reduced ZnO peak intensities, are seen. The broad peak for (002) is indicative of the smaller grain size, which is also confirmed by the Scherrer equation^28^ (Table 1).

**Table 1:** The crystallite size, d-spacing, plane (hkl), and morphologies of ZnO nanostructures and Ag.

Sample	Morphology	Plane (hkl)	d (Å)	Crystallite size (nm) (Scherrer)
100 wt% ZnO	Nanorods	(101)	2.47	38.60
95 wt% ZnO	Nanorods	(101)	2.47	39.23
90 wt% ZnO	Nanorods	(101)	2.47	32.35
70 wt% ZnO	Nanoparticles	(101)	2.47	24.83
50wt% ZnO	Nanoparticles	(101)	2.47	32.73
40wt% ZnO	Nanoparticles	(101)	2.47	21.43
5wt% Ag	Nanoparticles	(111)	2.35	60.20
10wt% Ag	Nanoparticles	(111)	2.35	74.09
30wt% Ag	Nanoparticles	(111)	2.35	68.66
50wt% Ag	Nanoparticles	(111)	2.35	72.26
MWCNTs	MWCNTs	(002)	3.43	4.66

The supplemental Table S1 reveals that the grain size for all the ZnO peak reflections decreases with increasing silver doping.

The lattice parameters for the hexagonal ZnO structure have been calculated using Bragg’s equation^29^:

1 $$2{d}_{(hkl)}sin\theta =n\lambda$$

Where *d* is the inter-planar distance, θ is the Bragg diffraction angle, n is the diffraction order (n = 1), and λ is the radiation wavelength (λ = 1.54 Å).

The lattice parameter “*a*” for ZnO is estimated from the (100) peak using the following equation^30^:

2 $$a=\frac{\lambda }{\sqrt{3} \,{\text{sin}}{\theta }_{(100)}}$$

On the other hand, the lattice constant “*c*” of the ZnO is obtained from the XRD peak belonging to (002) reflection using the following equation^30^:

3 $$c=\frac{\lambda }{{\text{sin}}{\theta }_{(002)}}$$

The lattice constants of the bulk ZnO based on the JCPD (no. 01-082-9744) are *a/b* = 3.2468 Å and c = 5.2019 Å. In the supplemental Table S2, we calculated the lattice parameters for pure and composited ZnO using Eqs. (2 and 3). Notably, as the concentration of Ag within the ZnO matrix increases, the cell parameters *a* and *c* also exhibit a corresponding increase. This alteration in the lattice parameters signifies a discernible deviation from the values observed in pure ZnO, possibly indicating the successful incorporation of Ag as a dopant into the ZnO structure.

**Figure 2:**
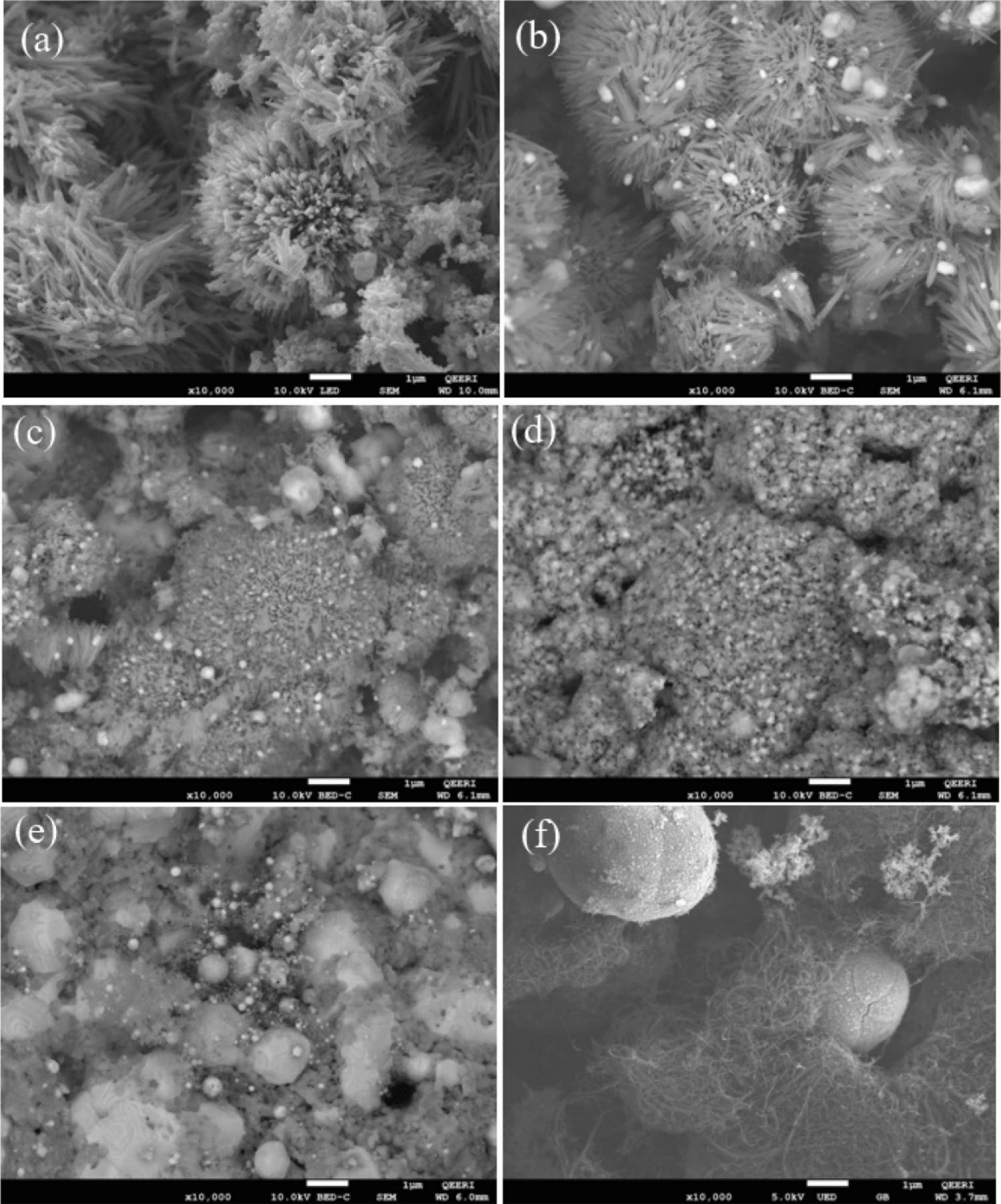
Low magnification (×10,000 magnification and 1 µm scale bar) SEM images of ZnO nanorods (**a**); 95 wt% ZnO + 5 wt% Ag (**b**); 90 wt% ZnO + 10 wt% Ag (**c**); 70 wt% ZnO + 30 wt% Ag (**d**); 50 wt% ZnO + 50 wt% Ag (**e**); and 50 wt% MWCNTs + 40 wt% ZnO + 10 wt% Ag (**f**).

**Figure 3:**
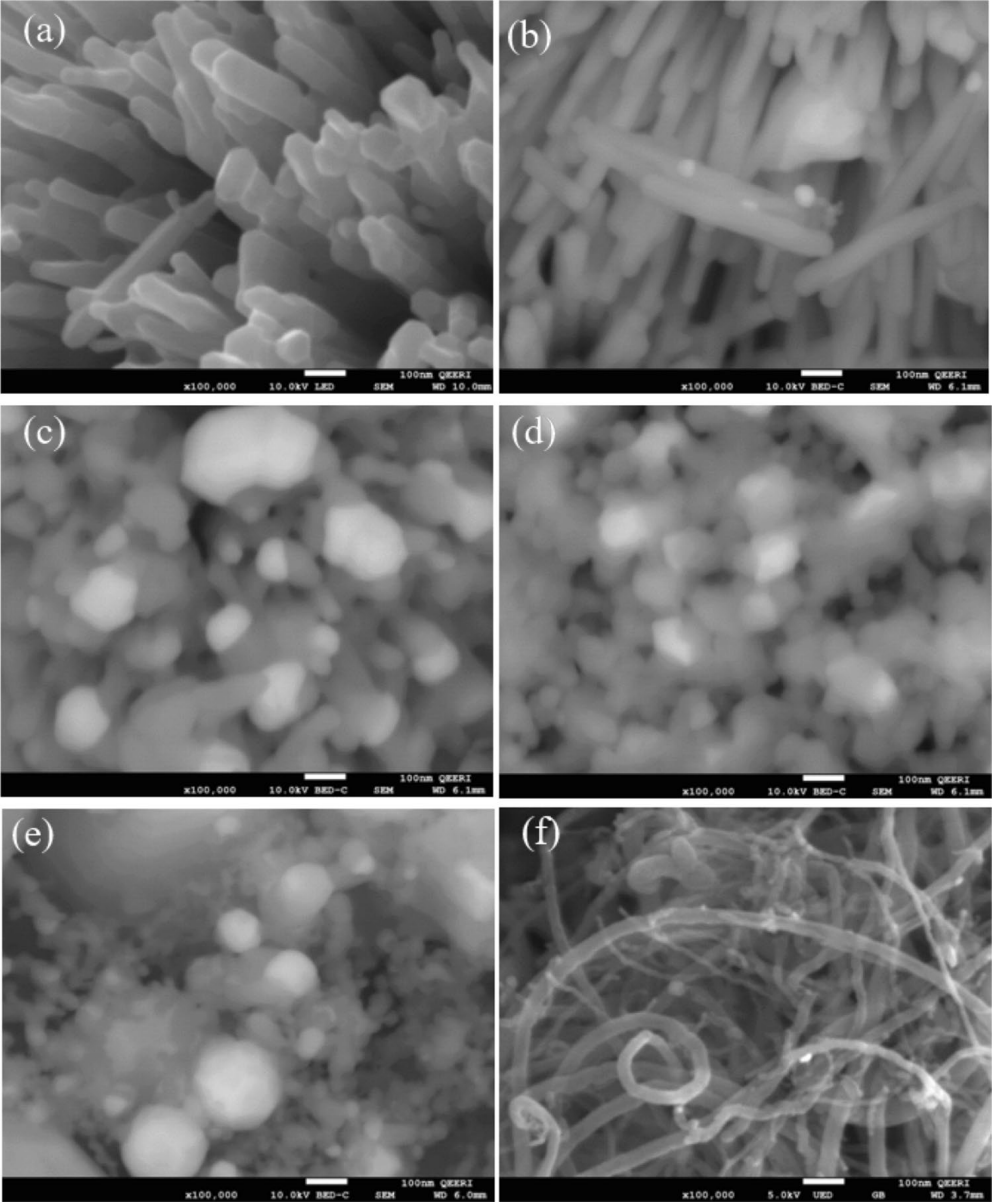
High magnification (×100,000 magnification and 100 nm scale bar) SEM images of ZnO nanorods (**a**); 95 wt% ZnO + 5 wt% Ag (**b**); 90 wt% ZnO + 10 wt% Ag (**c**); 70 wt% ZnO + 30 wt% Ag (**d**); 50 wt% ZnO + 50 wt% Ag (**e**) and 50 wt% MWCNTs + 40 wt% ZnO + 10 wt% Ag (**f**).

**Figure 4:**
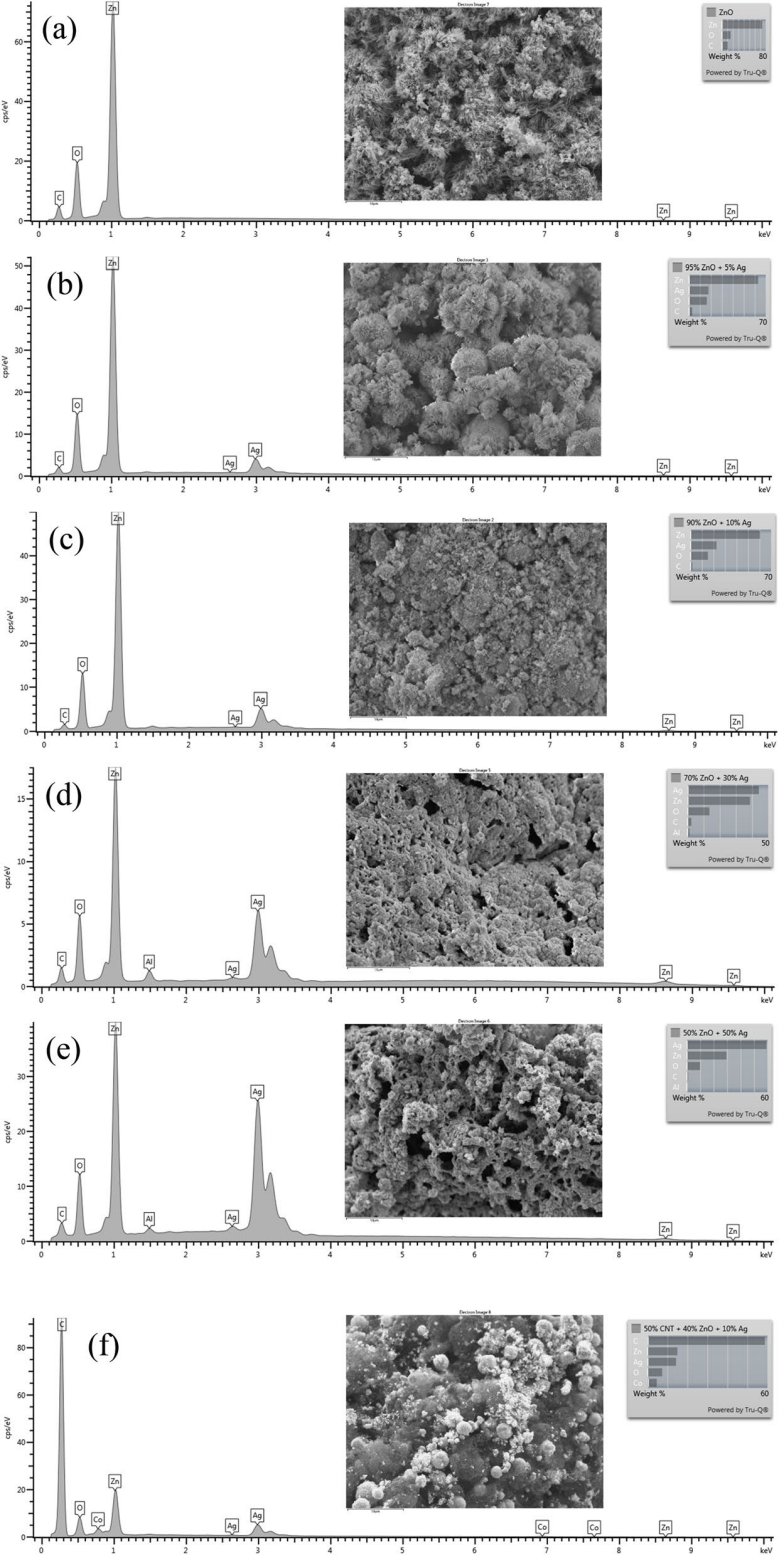
EDS analysis of (**a**) ZnO nanorods; (**b**) 95 wt% ZnO + 5 wt% Ag; (**c**) 90 wt% ZnO + 10 wt% Ag; (**d**) 70 wt% ZnO + 30 wt% Ag; (**e**) 50 wt% ZnO + 50 wt% Ag and (**f**) 50 wt% MWCNTs + 40 wt% ZnO + 10 wt% Ag.

**Figure 5:**
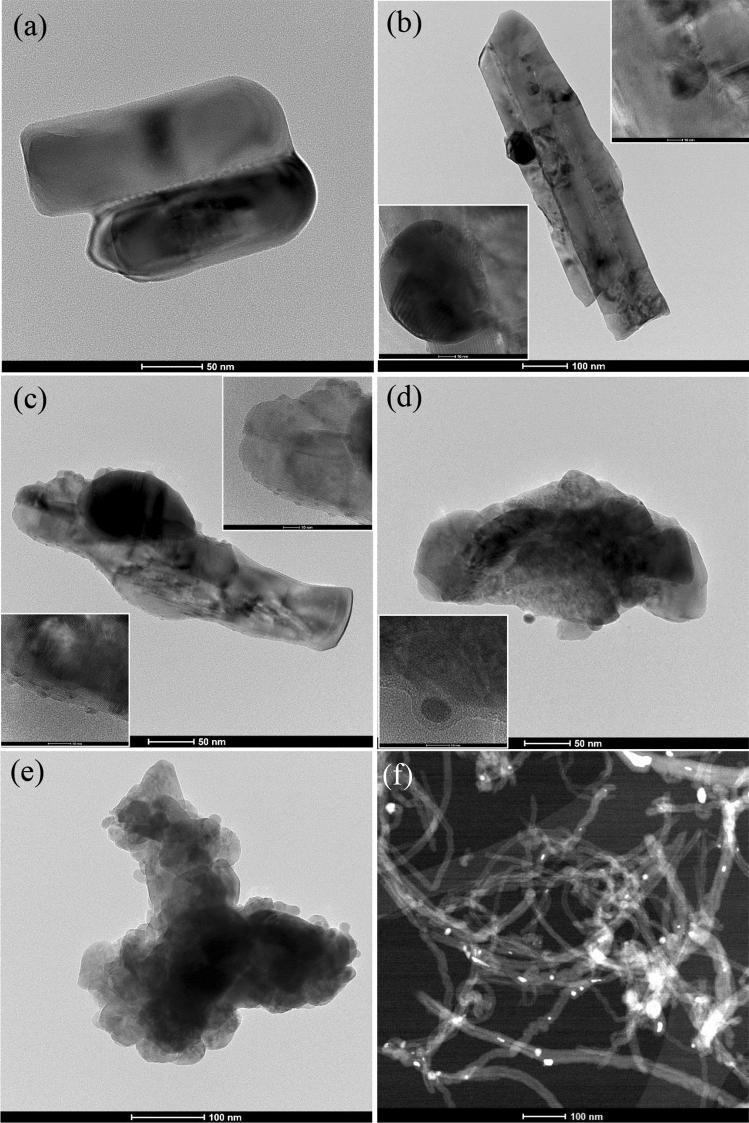
TEM images of ZnO nanorods (**a**); (**b**) 95 wt% ZnO + 5 wt% Ag; (**c**) 90 wt% ZnO + 10 wt% Ag; (**d**) 70 wt% ZnO + 30 wt% Ag; (**e**) 50 wt% ZnO + 50 wt% Ag and (**f**) 50 wt% MWCNTs + 40 wt% ZnO + 10 wt% Ag.

**Figure 6:**
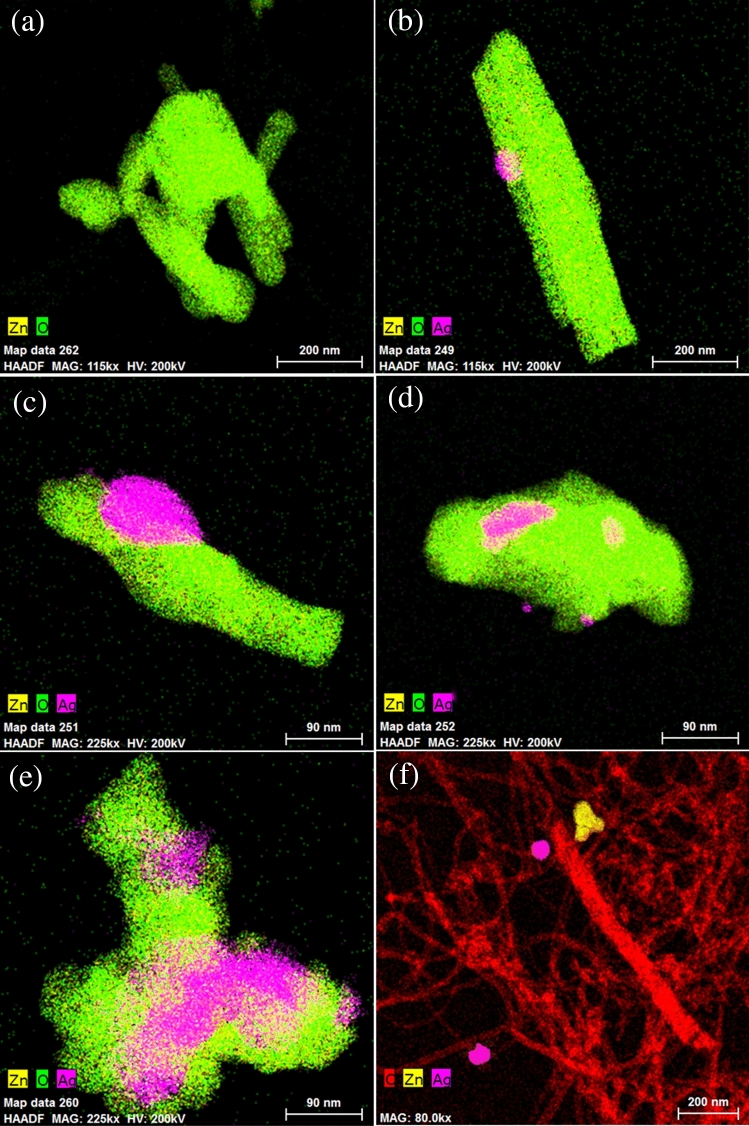
EDS mapping of (**a**) ZnO nanorods; (**b**) 95 wt% ZnO + 5 wt% Ag; (**c**) 90 wt% ZnO + 10 wt% Ag; (**d**) 70 wt% ZnO + 30 wt% Ag; (**e**) 50 wt% ZnO + 50 wt% Ag and (**f**) 50 wt% MWCNTs + 40 wt% ZnO + 10 wt% Ag.

Morphologies of the samples prepared via the simple impregnation–calcination method are studied by SEM imaging, as shown in Figs. 2a–f, 3a–f, and TEM imaging, as shown in Fig. 5. EDS analysis of the samples is shown in Fig. 4, while Fig. 6 shows the EDS mapping of synthesized materials. Figure 2a shows the low-magnification SEM image of ZnO nanorods, which appear like coral reefs. ZnO nanorods have diameters ranging from 30 to 100 nm, as shown in the high-magnification SEM image (Fig. 3a). Figure 2b displays the low magnification SEM image of ZnO nanorods doped with 5 wt% Ag nanoparticles found attached to the tips of ZnO nanorods. The Ag^+^ cations are possibly attracted to the negative growth unit ${{\text{Zn}}({\text{OH}})}_{4}^{2-}$ of ZnO or attracted by wurtzite ZnO polar surfaces, such as (000-1).

### Growth mechanism of the Ag-ZnO nanostructures

The growth mechanisms of ZnO- composited Ag and ZnO-Ag-MWCNT nanocomposites by one-step thermal decomposition of zinc acetate dihydrate and silver nitrate at 400 °C in air have been studied. The process begins with the mixing of zinc acetate dihydrate and silver nitrate with MWCNTs. The mixture is then heated to 400 °C, which causes the zinc acetate dihydrate and silver nitrate to decompose as shown in Eqs. (4) to (7). The zinc ions then react with oxygen in the air to form ZnO nanoparticles. The Ag ions, on the other hand, grow on the MWCNT's surface to form Ag nanoparticles. The ZnO and Ag nanoparticles are formed and grown using MWCNTs as a template and deionized water mixed with nitric acid as a medium. The MWCNTs might provide a large surface area for the ZnO and Ag nanoparticles to grow on, leading to the formation of ZnO-Ag-MWCNTs nanoparticles. A possible growth mechanism for the ZnO nanorods was previously described in detail elsewhere^31^. The possible chemical reactions to form ZnO and Ag nanostructures by thermal decomposition of zinc acetate dihydrate and silver nitrate, respectively, at 450 °C could be described as follows^32^:

4 $${\text{Zn}}{({{\text{CH}}}_{3}{\text{COO}})}_{2} \cdot 2{{\text{H}}}_{2}{\text{O}}\stackrel{\Delta }{\to } {\text{Zn}}{({{\text{CH}}}_{3}{\text{COO}})}_{2}+2{{\text{H}}}_{2}{\text{O}}\uparrow$$

5 $$4{\text{Zn}}{({{\text{CH}}}_{3}{\text{COO}})}_{2}\stackrel{\Delta }{\to } {{\text{Zn}}}_{4}{\text{O}}{({{\text{CH}}}_{3}{\text{COO}})}_{6}+ {({{\text{CH}}}_{3}{\text{CO}})}_{2}{\text{O}}\uparrow$$

6 $${{\text{Zn}}}_{4}{\text{O}}{({{\text{CH}}}_{3}{\text{COO}})}_{6}\stackrel{\Delta }{\to } 4{\text{ZnO}} ({\text{s}})+ 3{{\text{CH}}}_{3}{{\text{COCH}}}_{3}\uparrow +3{{\text{CO}}}_{2}\uparrow$$

7 $$2{{\text{AgNO}}}_{3}+ {3{\text{O}}}_{2}\stackrel{\Delta }{\to } 2{\text{Ag}}\left({\text{s}}\right)+{6{\text{NO}}}_{2}\uparrow$$

Typically, hexagonal ZnO is a polar crystal, which has positive polar planes (rich in Zn^+^ cations) and negative polar planes (rich in O^−^ ions). The polar zinc (0001) planes are less stable thermodynamically, causing growth in a particular direction to form nanorods^33^. The Ag^+^ cations are possibly attracted to the negative growth unit ${{\text{Zn}}({\text{OH}})}_{4}^{2-}$ of ZnO or attracted by wurtzite ZnO polar surfaces, such as (000-1). From Figs. 2 and 3, it can be noticed that the growth of zinc nanorods decreases with an increase in the percentage of silver, indicating that the silver nanoparticles are grown on ZnO surfaces. Therefore, Ag is likely limiting the growth of ZnO nanorods. The EDS shows that the particles are composed only of the elements Zn, O, and C.

Ag nanoparticle distribution on ZnO can be seen by using a Backscatter Electron (BSE) imaging detector. The BSE detector shows more contrast for the signal from Ag (47) compared to Zn (30)-O (8). Figures 2 and 3 show the low- and high-magnification SEM images, while Figs 5 and 6 display TEM images and EDS mapping of the prepared samples.

### Antibacterial activity of prepared materials

**Figure 7:**
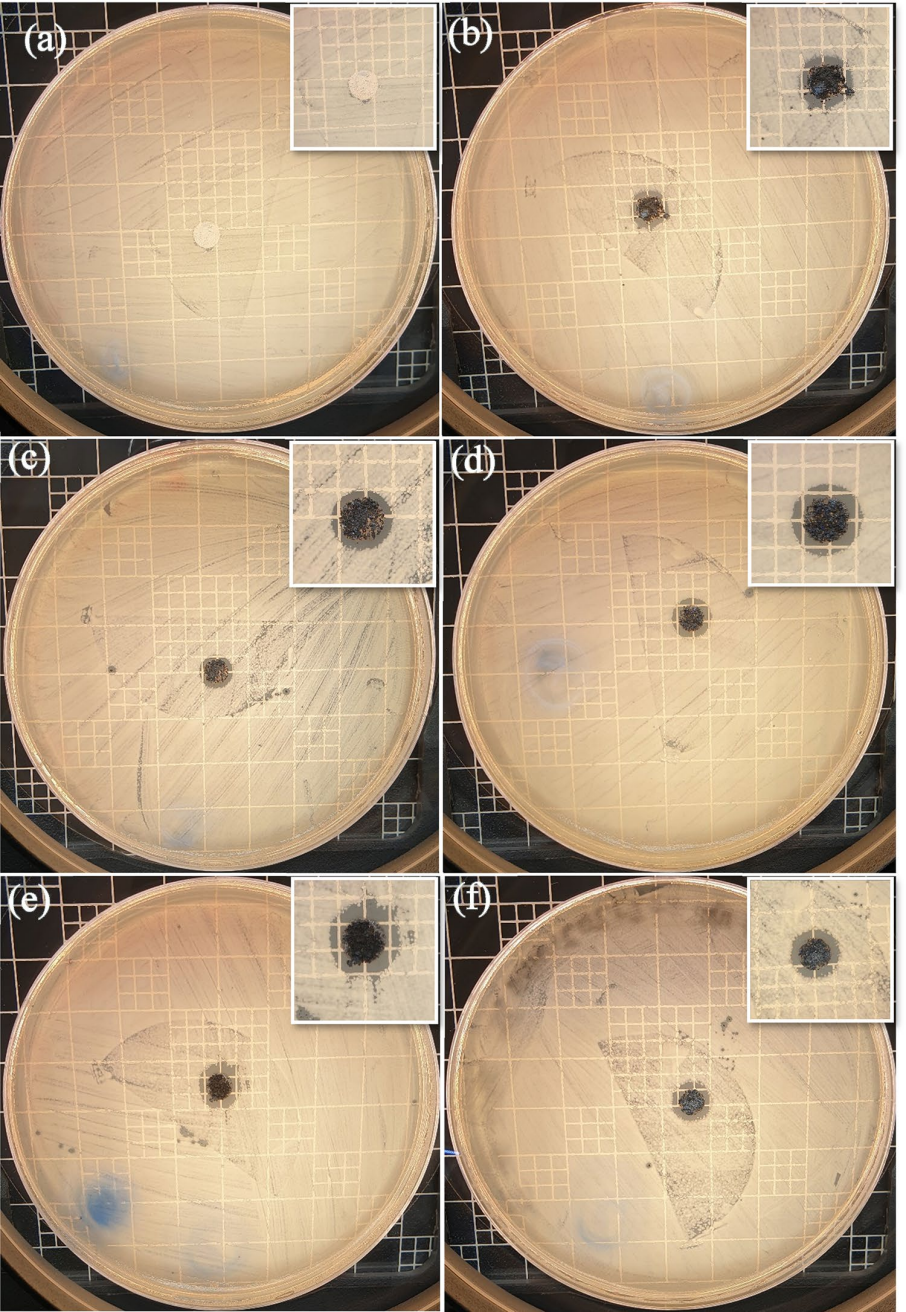
Antibacterial properties towards *E*. *coli* bacteria of (**a**) ZnO nanorods; (**b**) 95 wt% ZnO + 5 wt% Ag; (**c**) 90 wt% ZnO + 10 wt% Ag; (**d**) 70 wt% ZnO + 30 wt% Ag; (**e**) 50 wt% ZnO + 50 wt% Ag and (**f**) 50 wt% MWCNTs + 40 wt% ZnO + 10 wt% Ag.

**Figure 8:**
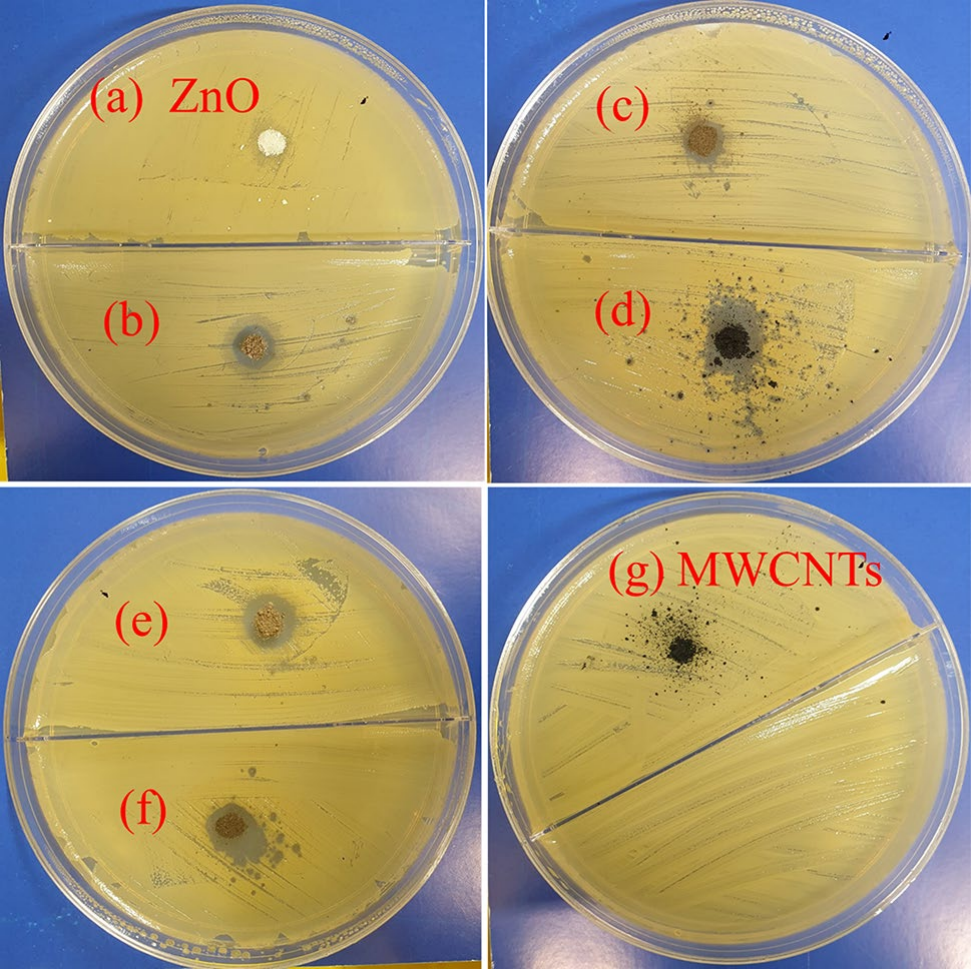
Zone of inhibition for the synthesized materials against gram-positive bacteria (*Staphylococcus aureus*).

Antibacterial properties of raw ZnO and composited ZnO nanostructures with silver and multi-walled carbon nanotubes towards gram-negative (*E. coli*) bacteria have been studied as described in “Antibacterial activity of prepared materials” section, and the inhibition zone data are displayed in Fig. 7 and Table 2. On the other hand, Fig. 8 shows antibacterial properties towards gram-positive bacteria (*S. aureus*) of (a) pure ZnO, (b) 95 wt% ZnO + 5 wt% Ag, (c) 90 wt% ZnO + 10 wt% Ag, (d)50 wt% MWCNTs + 40 wt% ZnO + 10 wt% Ag, (e) 70 wt% ZnO + 30 wt% Ag, (f) 50 wt% ZnO + 50 wt% Ag and (g) is raw MWCNTs. The control images of *S. aureus* and *E. coli* are displayed in Fig. S1.

**Table 2:** Inhibition zones of the prepared composite materials towards *E*. *coli* and *S. aureus* bacteria.

Sample	Zones of inhibition for *E. coli* (mm) ± 1	Zones of inhibition for *S. aureus* (mm) ± 1
ZnO	0	0
ZnO + 5% Ag	7.4	9
ZnO + 10% Ag	7.6	8
ZnO + 30% Ag	9.1	13
ZnO + 50% Ag	9.2	14
MWCNTs + 40% ZnO + 10% Ag	8.5	22
MWCNTs	0	0

The produced ZnO nanorods did not exhibit a clear inhibition zone with the *E. coli* and *S. aureus* bacteria, as shown in Figs. 7 and 8. This finding, while unexpected, could be explained by the low quantity of ZnO material used for testing. Previously it was reported that the antibacterial activity of ZnO against *E. coli* is highly loading-dependent^34^. On the other hand, Fig. 7 and Table 2 show clear zones of inhibition for ZnO composited with silver and MWCNTs against *E. coli* bacteria. However, the strong zones of inhibition for ZnO nanorods composited with silver and MWCNTs against *S. aureus* bacteria were shown in Fig. 8 and Table 2. It seems that ZnO composite materials possess stronger antimicrobial action against *S. aureus* bacteria than *E. coli bacteria*. Although S. aureus bacteria have a much thicker outer wall than *E. coli bacteria*. However, *E. coli bacteria* are generally more resistant to antibiotics due to the presence of an outer membrane (peptidoglycan-porin proteins) that protects the cell wall. This membrane is composed of lipopolysaccharides (LPS), which create a barrier that prevents many antibiotics from penetrating the cell wall^35,36^.

The diameter of the inhibition zone increases up to 9.2 mm in diameter for *E. coli* and up to 14 mm for *S. aureus* with an increase in Ag loading in the composite materials. The large inhibition zones demonstrated are obviously due to the bactericidal effect of doped Ag on *E. coli* and *S. aureus* cells. Despite the fact that antibacterial properties of silver-containing materials is of high research interest because of the low toxicity of silver to humans^37^, however, mechanisms that are responsible for the bactericidal action of these materials have not been precisely established yet. Some studies reported that silver nanoparticles can adhere and penetrate into *E. coli* bacteria, thus causing damage to the bacterial cell^38^. It was also suggested that silver ions can bind to some bacterial enzymes, and by breaking the respiratory chain, cause the death of the bacterial cell^39^. In general, it was indicated that typical mechanisms of bactericidal action of silver doped materials might include sorption of silver ions by a bacterial cell with subsequent disruption of DNA replication or generation of reactive oxygen species in the presence of silver and free oxygen, which can attack and damage the outer membrane of a bacterial cell and lead to its death^40^. Ag and Zn ions are present, which strongly implies that these ions either activate intracellular reactive oxygen species or significantly interact electrostatically with the negatively charged bacterial cell membrane, causing bacterial cell death. Disrupting membrane function and oxidizing biomacromolecules had a considerable impact on the antibacterial action of ZnO-Ag nanocomposites. Kang et al.^41^ reported SWCNTs as an antibacterial agent against *E. coli* and SWCNTs are significantly more toxic to bacteria than MWCNTs^26^. The highest observed zone of inhibition value was 22 mm for the hybrid sample (50 wt% MWCNTs + 40 wt% ZnO + 10 wt% Ag), as shown in Fig. 8d. Some antimicrobial composite materials similar to this work are summarized in Table 3.

**Table 3:** Antimicrobial composite materials and their preparation methods and effects on inhibition zones for *E. coli* and *S. aureus* bacteria.

Antimicrobial composite materials	Preparation methods	Zones of inhibition for *E. coli* (mm)	Zones of inhibition for *S. aureus* (mm)	Reference
Ag doped ZnO-MWCNT	Spray pyrolysis	–	18.62	^42^
Ag/0.5 g ZnO-clay	Microwave-assisted synthesis	8	–	^43^
Ag-ZnO/activated carbon	Co-precipitation	8	12	^44^
ZnO doped Ag	Electrospinning	9.7	8.3	^45^
Ag-loaded ZnO	Sol–gel	8	4	^46^
ZnO-Ag heterostructure nanoparticles	Precipitation	–	9.1	^47^
ZnO-Ag nanocomposites	Simple impregnation–calcination	9.2	14	This study
ZnO-Ag-MWCNTs composites	Simple impregnation–calcination	8.5	22	This study

**Figure 9:**
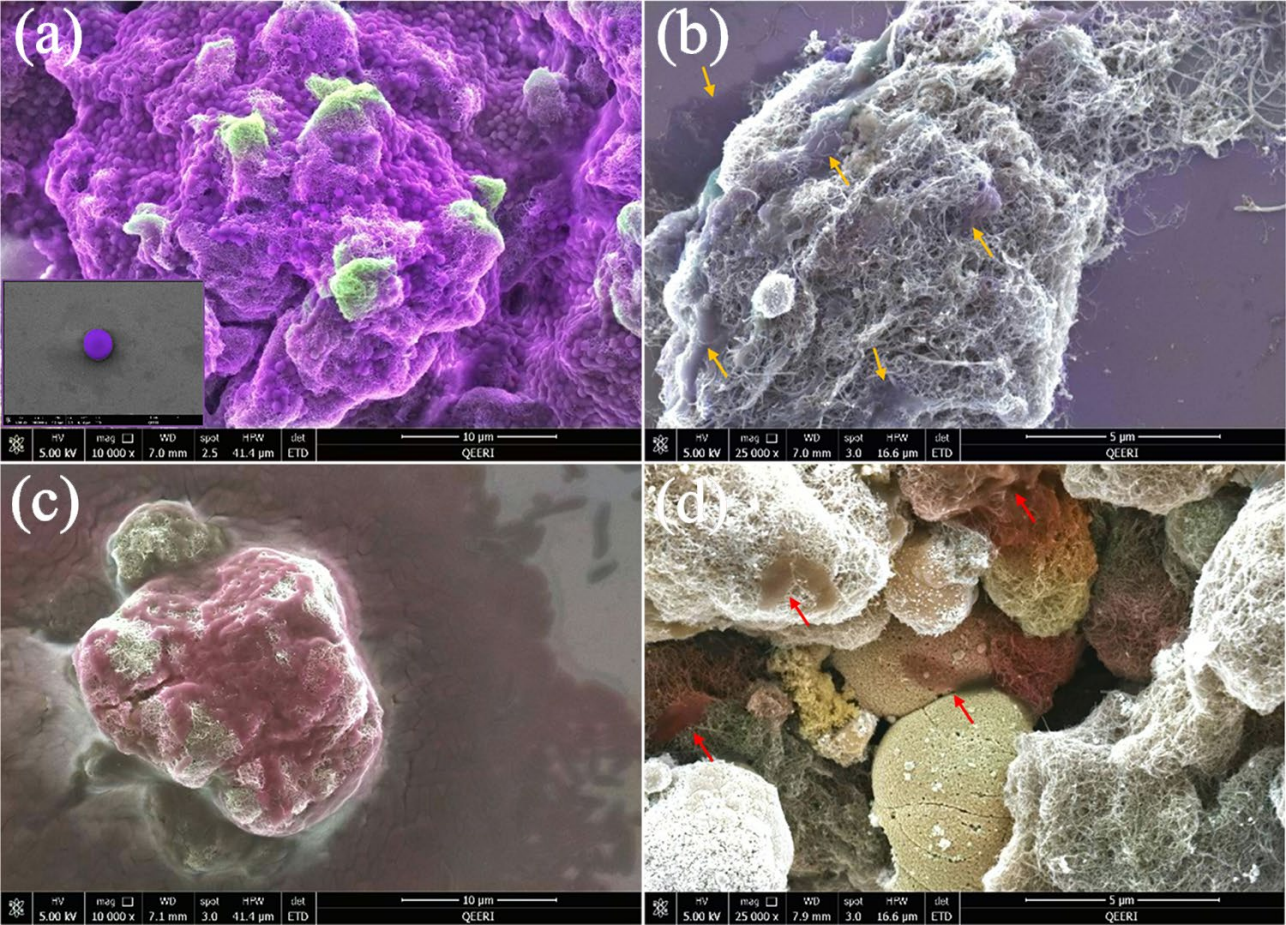
SEM images of inhibition zones: (**a**) raw MWCNTs with *S. aureus*; (**b**) 50 wt% MWCNTs + 40 wt% ZnO + 10 wt% Ag with *S. aureus*; (**c**) raw MWCNTs with *E. coli;* and (**d**) 50 wt% MWCNTs + 40 wt% ZnO + 10 wt% Ag with *E. coli.* Inserted in (**a**) is an SEM image of a healthy *S. aureus* single cell.

**Figure 10:**
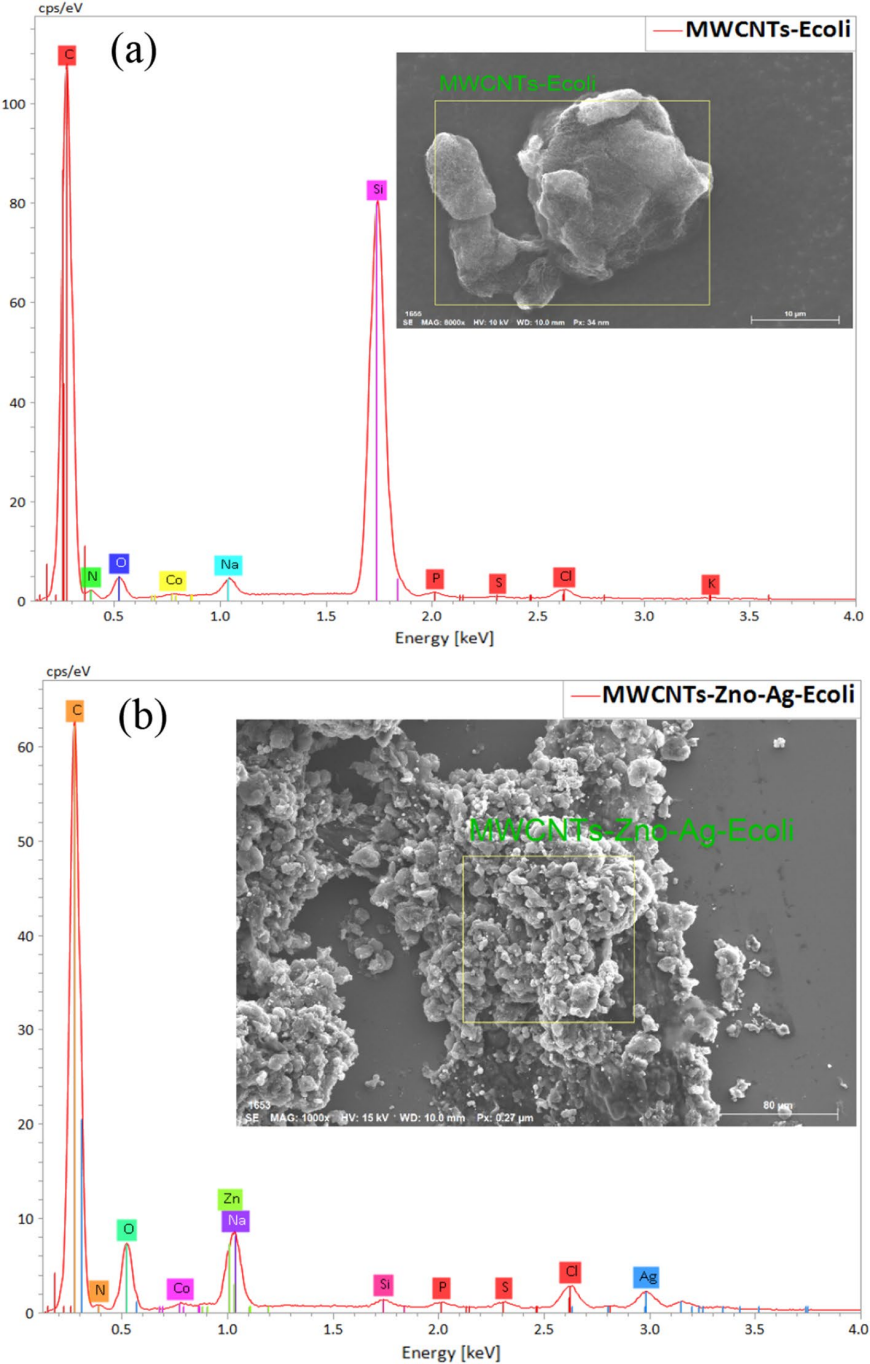
EDS analysis of raw MWCNTs with *E. coli* (**a**)*;* and 50 wt% MWCNTs + 40 wt% ZnO + 10 wt% Ag with *E. coli* (**b**).

To find out the effect of the raw and composited materials on the cell morphology of positive and negative bacteria, the zones of inhibition with the raw and composited materials were analyzed using SEM, as shown in Figs. 9 and 10.

**Figure 11:**
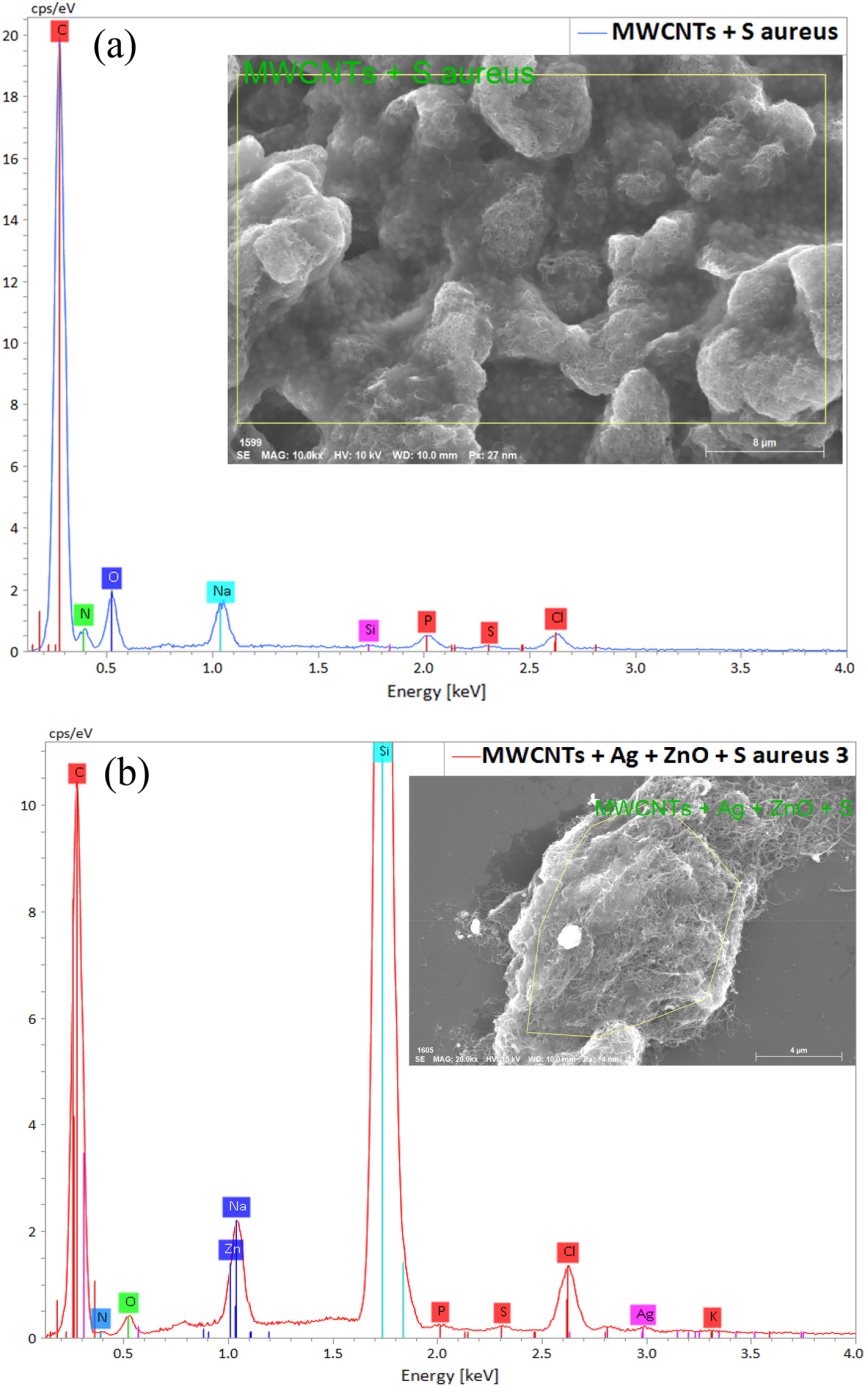
EDS analysis of raw MWCNTs with *S. aureus* (**a**)*;* and 50 wt% MWCNTs + 40 wt% ZnO + 10 wt% Ag with *S. aureus* (**b**).

Figure 9 shows the effect of raw MWCNTs and MWCNTs composited with ZnO and Ag on the cell morphology of *S. aureus* (a and b) and *E. coli* (c and d). In Fig. 9a, *S. aureus* cells with distinct, compact, and undamaged cell morphology completely cover raw MWCNTs. While the MWCNTs composited with ZnO and Ag sample has a strong effect on *S. aureus* cells, as shown in Fig. 9b. In Fig. 9b, the yellow arrows indicate cell membrane disruption, cell deformation, and leakage as compared to Fig. 9a. *E. coli* cells are affected in the same way as shown in Fig. 9c and d. The EDS analysis that corresponds to Fig. 9 is shown in Figs. 10 and 11.

### FTIR study

**Figure 12:**
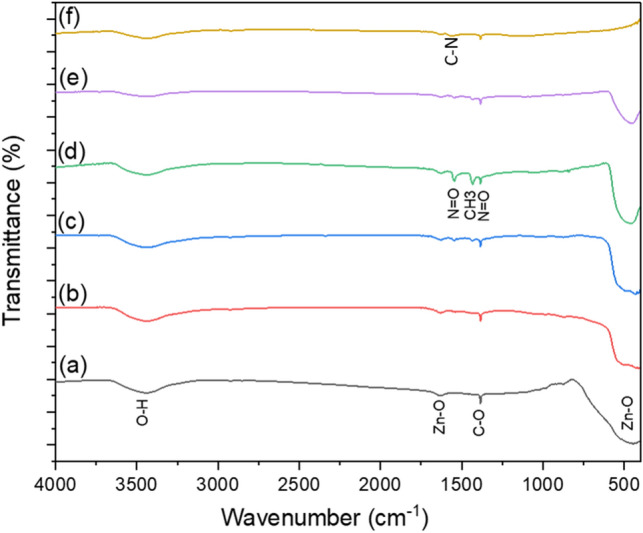
FTIR spectra of (**a**) ZnO nanorods; (**b**) 95 wt% ZnO + 5 wt% Ag; (**c**) 90 wt% ZnO + 10 wt% Ag; (**d**) 70 wt% ZnO + 30 wt% Ag; (**e**) 50 wt% ZnO + 50 wt% Ag and (**f**) 50 wt% MWCNTs + 40 wt% ZnO + 10 wt% Ag.

The purpose of the FTIR investigation was to analyze the composition of the prepared composite materials and any potential interactions between them, to discover any functional groups adsorbing species onto the surface of the composite materials as prepared, and to find any molecular impurities. Three main absorption bands are visible in all FTIR spectra centered at around 3440, 1635, and 1385 cm^−1^ (Fig. 12), which can be attributed to the stretching of intermolecular O–H and hydroxyl groups, Zn–O stretching vibration and asymmetric C–O, respectively^48–50^. Strong broadband is observed at low wave numbers centered at 460 cm^−1^ and is due to Zn–O stretching vibration^51^. The bands at 1445 and 1540 cm^−1^ are assigned for CH_3_ symmetrical bending vibration and asymmetric stretch of N=O nitroso^52^, respectively. It should be noted that the symmetric stretch of N=O is overlapping with the C–O band at 1385 cm^−1^ (Fig. 12b–e). The peak at 1448 cm^−1^ could be assigned to C–N stretching of amide groups^53^ (Fig. 12f). The absence of bands in the FTIR spectra associated with AgO or Ag_2_O_3_ indicates that Ag must be in its metallic state^54^, as conformed by XRD (Fig. 1).

## Conclusions

ZnO composited with Ag and MWCNTs were prepared by a simple impregnation–calcination method. It should be highlighted that there is no need for additional additives such as organic solvents, oxidation agents, or reducing agents to synthesize ZnO composited with Ag and MWCNTs by employing this simple, time- and cost-efficient preparation method. The produced nanocomposite materials' morphology, particle size, structure, and chemical composition were examined using XRD, TEM, SEM, and EDS techniques. The mechanism of formation of ZnO-based nanocomposites was also discussed. The antibacterial effect of the composite materials was studied against *E. coli and S. aureus* by measuring the bacterial inhibition zone using the agar-well diffusion method. Using composited ZnO composite materials, significant inhibition zones up to 9.2 mm in diameter for *E. coli* and up to 22 mm for *S. aureus* were detected. The zones of inhibition with the raw and composited materials were investigated using SEM to determine the impact of the nanocomposite materials on the cell morphology of *E. coli* and *S. aureus*. These findings prove the strong antibacterial properties of the synthesized nanocomposite materials, which can be promising for their use as biocidal agents in water treatment applications. A minimum inhibitory concentration (MIC) assay of the produced nanocomposite materials will be conducted in subsequent work.

### Supplementary Information

Supplementary Information.

## Data Availability

The datasets used and/or analyzed during the current study available from the corresponding author on reasonable request.
