# Supplementary material for: Antimicrobial activity of ZnO-Ag-MWCNTs nanocomposites prepared by a simple impregnation–calcination method
Source: Sci Rep. 2023 Dec 5;13:21418. doi: 10.1038/s41598-023-48831-w (PMC10695929; doi:10.1038/s41598-023-48831-w)
Supplement: Supplementary file 1 — Supplementary Information. [file 41598_2023_48831_MOESM1_ESM.docx]

**Supplementary material**

**Antimicrobial activity of ZnO-Ag-MWCNTs nanocomposites prepared by a simple impregnation-calcination method**

Rashad Al-Gaashani^⁎,a^, Mujaheed Pasha^b^, Khadeeja Abdul Jabbar^a^, Akshath R. Shetty^b^, Hussein Baqiah^c^, Said Mansour^b^, Viktor Kochkodan^a^, Jenny Lawler^a^

*^a^Qatar Environment and Energy Research Institute (QEERI), Hamad Bin Khalifa University (HBKU), Qatar Foundation, 34110 Doha, Qatar*

*^b^HBKU Core Labs, Hamad Bin Khalifa University, Qatar Foundation, Doha, Qatar*

*^c^Shandong Key Laboratory of Biophysics, Institute of Biophysics, Dezhou University, No.566 University Rd. West, Dezhou, Shandong, China*

****Corresponding author.*** *Tel: 0097430571456. E-mail:* [*ralgaashani@hbku.edu.qa*](about:blank)

Table S1: The crystallite size of various (hkl) planes corresponding to ZnO peaks

| Sample | Crystallite size (nm) (Scherrer) (101) | Crystallite size (nm) (Scherrer) (100) | Crystallite size (nm) (Scherrer) (002) | Crystallite size (nm) (Scherrer) (103) | Crystallite size (nm) (Scherrer) (102) |
| --- | --- | --- | --- | --- | --- |
| 100wt.% ZnO | 38.60 | 40.71 | 42.56 | 29.22 | 31.92 |
| 95 wt.% ZnO | 39.23 | 38.02 | 56.77 | 36.34 | 37.37 |
| 90wt.% ZnO | 32.35 | 32.69 | 38.22 | 25.77 | 27.37 |
| 70wt.% ZnO | 24.83 | 30.42 | 30.60 | 22.69 | 22.03 |
| 50wt.% ZnO | 32.73 | 38.64 | 34.06 | 27.55 | 27.48 |
| 40wt.% ZnO | 21.43 | 23.21 | 27.97 | 16.76 | 20.22 |

Table S2: The lattice parameters for pure and doped ZnO

| Sample | C (Å) | A (Å) |
| --- | --- | --- |
| 100 wt.% ZnO | 5.193785 | 3.241373 |
| 95 wt.% ZnO + 5 wt.% Ag | 5.193201 | 3.241175 |
| 90 wt.% ZnO + 10 wt.% Ag | 5.192909 | 3.240878 |
| 70 wt.% ZnO + 30 wt.% Ag | 5.196852 | 3.243259 |
| 50 wt.% ZnO + 50 wt.% Ag | 5.202706 | 3.24624 |
| 40 wt.% MWCNTs + 50 wt.% ZnO + 10 wt.% Ag | 5.198022 | 3.245444 |

(a)


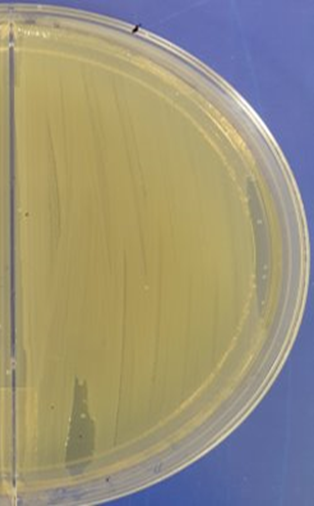

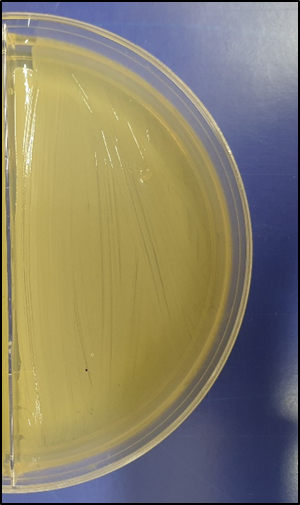


(b)

Fig. S1. The control images of *E. coli* (a) and *S. aureus* (b).
